# P90 Comparative clinical outcomes and prognostic determinants in neonatal sepsis: insights from a multicentre prospective cohort study

**DOI:** 10.1093/jacamr/dlag102.096

**Published:** 2026-06-26

**Authors:** Ifeyinwa Nwafia, Chioma Achi, Aminu Aliyu, Nubwa Medugu, Yahaya Mohammed, Oduyebo Oyinlola, Khadija Abduraheem, Kirsty Sands, Katy Thomson, Kenneth Iregbu, Timothy R Walsh

**Affiliations:** Department of Medical Microbiology, University of Nigeria Teaching Hospital, Enugu, Nigeria; Department of Infectious Disease Epidemiology, London School of Hygiene and Tropical Medicine, Keppel Street, London, UK; Ineos Oxford Institute for Antimicrobial Research, Department of Biology, University of Oxford, Oxford, UK; Department of Medical Microbiology, Aminu Kano Teaching Hospital, Kano, Nigeria; Department of Medical Microbiology, National Hospital, Abuja, Nigeria; Department of Medical Microbiology, Usman Danfodiyo University Teaching Hospital, Sokoto, Nigeria; Department of Medical Microbiology, Lagos University Teaching Hospital, Lagos, Nigeria; Department of Medical Microbiology, National Hospital, Abuja, Nigeria; Ineos Oxford Institute for Antimicrobial Research, Department of Biology, University of Oxford, Oxford, UK; Ineos Oxford Institute for Antimicrobial Research, Department of Biology, University of Oxford, Oxford, UK; Department of Medical Microbiology, National Hospital, Abuja, Nigeria; Ineos Oxford Institute for Antimicrobial Research, Department of Biology, University of Oxford, Oxford, UK

## Abstract

**Objectives:**

To determine the prevalence of clinically diagnosed sepsis, compare mortality rates among neonates with pathogen-confirmed sepsis, identify prognostic factors associated with mortality in neonatal sepsis, and provide evidence to inform improved infection management and antibiotic stewardship in neonatal units.

**Methods:**

We conducted a 19-month prospective cohort study in five Nigerian tertiary hospitals: National Hospital Abuja, University of Nigeria Teaching Hospital Enugu, Aminu Kano Teaching Hospital Kano, Usman Danfodiyo University Teaching Hospital Sokoto, and Massey Street Children/Lagos Island Maternity Hospital, Lagos. Neonates with suspected bloodstream infection (BSI) were consecutively enrolled during the period. Blood cultures were processed using automated BacT/Alert equipment, and antimicrobial susceptibility testing was interpreted with EUCAST guidelines. The patients were followed up until discharge (alive or dead) or for a period of 28 days, to determine mortality outcome.

**Results:**

A total of 15 932 neonates were recruited during the study period, of whom 3599 were clinically diagnosed with BSI. Among those clinically diagnosed, 1301 (36.1%) were pathogen-confirmed BSI. Of these confirmed infections, 884 (67.9%) were caused by Gram-positive bacteria, predominantly *Staphylococcus aureus*, 372 (28.6%) by Gram-negative bacteria, predominantly *Klebsiella* species, and 45 (3.5%) by *Candida* species. The overall mortality rate in pathogen-confirmed BSI was 13.5% (*n*=176/1301), which was significantly higher for Gram-negative bacteria (19.1%, *n*=71/372) and *Candida* spp (17.8%, *n*=8/45), compared to Gram-positive (10.9%, *n*=97/884) bacteria (*x*^2^=15.452, *P*=0.0004). Compared with neonates without BSI and those with clinically diagnosed but pathogen-unconfirmed BSI, neonates with fungal BSI demonstrated approximately five-fold increased risk of mortality (OR 5.4940; 95% CI 2.5465–11.8529; *P<*0.0001), 6-fold increased mortality risk for Gram-negative BSI (OR 5.9937; 95% CI 4.5655–7.8685; *P<*0.0001) and threefold increased mortality risk for Gram-positive BSI (OR 3.1318; 95% CI 2.4949-3.9313; *P<*0.0001). Regarding prognostic determinants, gestational age <28 weeks was a significant predictor of mortality among neonates with Gram-negative BSI (OR=2.9949, 95% CI=1.0299–8.7086, *P*=0.0440). At the same time, female sex was a significant predictor of mortality in neonates with fungal BSI (OR=10.2667, 1.1425–92.2598, *P*=0.0376). None of the evaluated risk factors was significantly associated with mortality in neonates with Gram-positive BSI.

**Conclusions:**

Neonates with Gram-negative and fungal BSIs experience substantially higher mortality compared to those with Gram-positive infections. These findings underscore the need for rapid diagnosis, optimized empirical therapy, particularly for treating fungal sepsis, and strengthened infection prevention and control practices in neonatal care units in Nigeria.

